# Blunt trauma as a suspected cause of delayed constrictive pericarditis: a case report

**DOI:** 10.1186/1752-1947-5-76

**Published:** 2011-02-23

**Authors:** Eric M Anderson, Dawn E Jaroszewski, Francisco A Arabia

**Affiliations:** 1 Department of Cardiothoracic Surgery; Mayo Clinic Arizona; 5777 East Mayo Boulevard; Phoenix, Arizona 85054, USA

## Abstract

**Introduction:**

Constrictive pericarditis is a heterogeneous disease with many causes. Traumatic hemopericardium is an uncommon initiating cause. We report the case of a man developing constrictive pericarditis after blunt chest trauma, in order to highlight an approach to diagnosing the condition and to raise awareness of the possibility of this condition developing after blunt trauma.

**Case presentation:**

A 72-year-old Caucasian man presented initially to our outpatient clinic with a one-year history of progressively worsening dyspnea, and recent onset of edema of the legs. He was later taken to the emergency department and admitted to hospital. He had previously received unsuccessful treatment from his local primary physicians for suspected respiratory disorder and cellulitis of his legs. Echocardiography showed evidence of pericardial constriction, and computed tomography revealed nodular, lobulated thickening of the pericardium and pleura bilaterally. Interventional biopsies were taken, but gave inconclusive results. Thus, as pericarditis and/or advanced malignancy were suspected, diagnostic video-assisted thoracoscopic surgery was performed to take biopsies from the abnormal lung and pericardial tissue. Examination of these supported the diagnosis of pericarditis, as acute and chronic inflammation and fibrous thickening were found, with no evidence of malignancy. Our patient underwent cardiac catheterization, which revealed three-vessel coronary artery disease. Emergency total pericardiectomy and coronary bypass were performed. Having excluded other common initiating factors, we considered that a blunt trauma that our patient had previously sustained to his chest was the potential cause of the constrictive pericarditis.

**Conclusion:**

This was an interesting case of blunt chest trauma followed by progressive pericardial and pleural thickening. Subsequent development of chronic constrictive pericarditis occurred, requiring treatment by surgical pericardiectomy, as the clinical course of constrictive pericarditis is usually progressive without surgical intervention. Diagnosis of constrictive pericarditis remains challenging. Although uncommon, blunt trauma should be considered as a possible initiating cause. Delayed presentation of constrictive pericarditis should also be considered as a possible morbidity in a patient who has sustained blunt chest trauma. Our case also highlights the importance of performing echocardiography promptly in patients experiencing ongoing symptoms of congestive heart failure to allow earlier diagnosis of constrictive pericarditis or other cardiac disorders, and avoid unnecessary treatments.

## Introduction

Constrictive pericarditis (CP) is a heterogeneous disease with many causes [1-4]. CP develops when progressive inflammation and fibrosis of the pericardium compress the myocardium, and impair normal filling of the ventricles. It is characterized by clinical signs of right heart failure subsequent to loss of pericardial compliance. Whereas in the past, tuberculosis was the prevalent cause of the disease, cardiac surgery and idiopathic pericardial constriction are now the most common causative factors [2,3]. CP is also caused by viral, bacterial or fungal infection, uremia, autoimmune disease, and inflammatory reaction to a foreign body. Traumatic hemopericardium is an additional yet uncommon initiating cause [5-8].

This case report highlights an approach to diagnosing constrictive pericarditis and aims to raise awareness of the possibility of this condition developing after blunt trauma.

## Case presentation

A 72-year-old Caucasian man presented to his local primary care physician with a one-year history of worsening dyspnea on exertion, along with edema of the legs. He reported recent paroxysmal nocturnal dyspnea and orthopnea, which required him to sleep in a reclining chair and an inability to walk more than a few steps without becoming considerably short of breath. He had no known history of coronary artery disease, and was not experiencing chest pain. Multiple tests for cardiac enzymes were negative. Echocardiograms performed eight months earlier showed mild dilation and hypokinesis of the right ventricle. Previous treatments for suspected obstructive lung disease and antibiotics for erythema and the leg edema had proved ineffective. The edema could not be attributed to deep vein thrombosis or to any marked obstructive pathology in the lungs, abdomen or pelvis.

Our patient was referred to our institution for a second opinion. On physical examination at rest, his temperature was 37°C; blood pressure 126/75 mm Hg, heart rate 95 beats/minute, respiration rate 20 breaths/minute, and oxygen saturation 91% on room air. During a visit to our outpatient clinic, our patient appeared cyanotic, and was taken to the emergency department for evaluation of his hypoxia. With ambulation, his oxygen saturation dropped to 87%, and he was later admitted to hospital.

**Figure 1:**
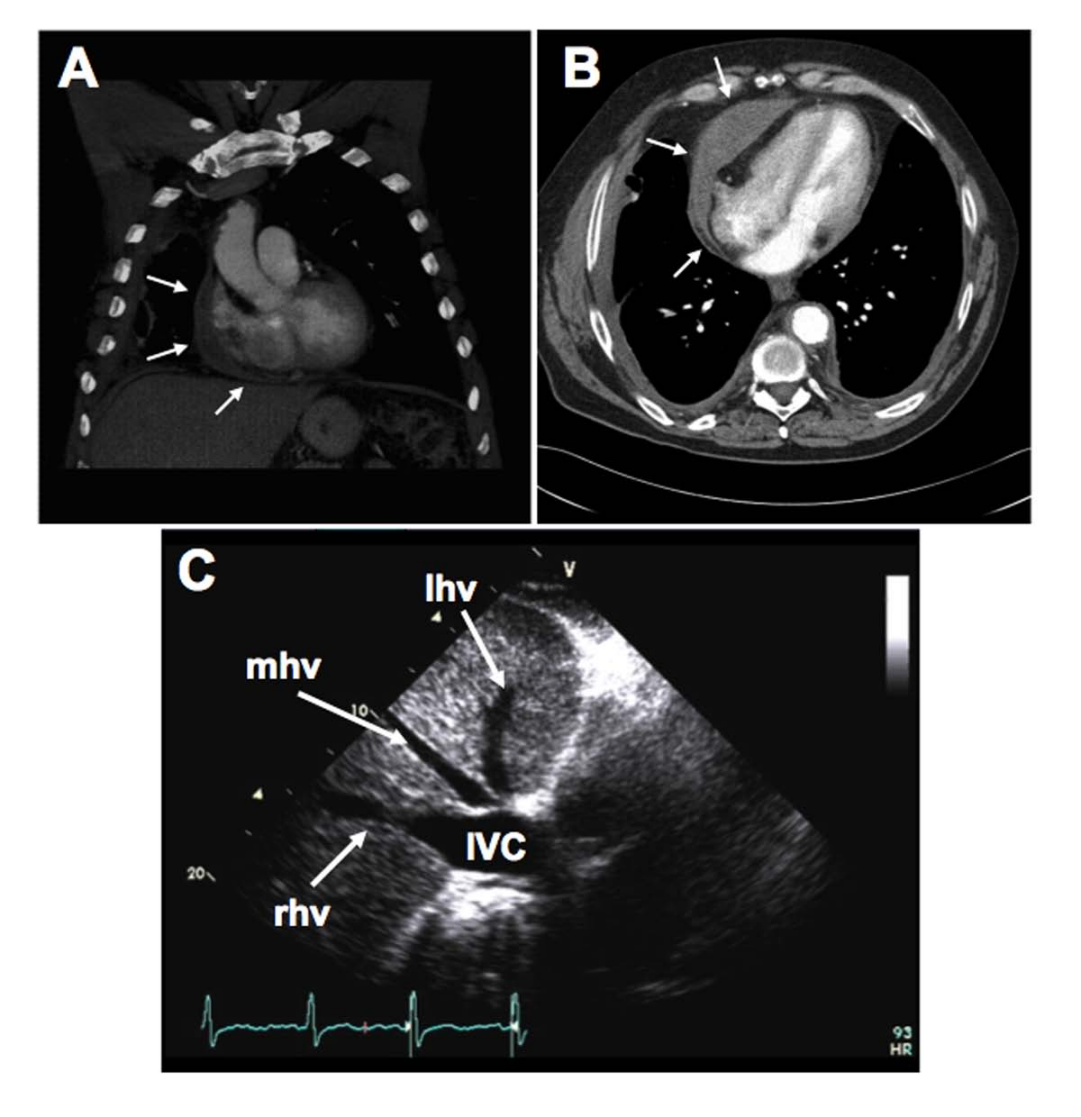
**Computed tomography (CT) scan identifying pericardial thickening and echocardiogram showing dilated intrahepatic vein and inferior vena cava**: (a,b) CT axial and coronal views of pericardial and pleural thickening. Arrows point to areas of thickened pleura and pericardium. (c) Transthoracic echocardiogram (TTE) showing dilated intrahepatic vein and inferior vena cava (IVC). The terms lhv, mhv, and rhv correspond to left, middle, and right hepatic veins, respectively.

**Figure 2:**
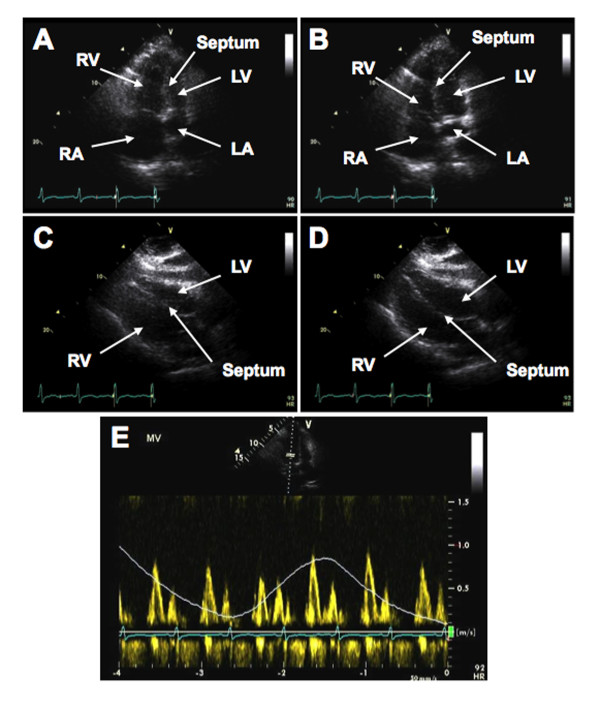
**Transthoracic echocardiogram (TTE) showing abnormal ventricular and interventricular movement and mitral flow**: TTE showing (a,b) marked interventricular movement; (c,d) restrictive movement of lateral ventricular walls with septal bounce; (e) tricuspid and mitral flow with inspiration and expiration. RA, LA, RV and LV correspond to the right and left atria and ventricles, respectively.

Extensive examinations were performed. Electrocardiography showed left atrial enlargement and non-specific T-wave abnormalities. Computed tomography (CT) revealed nodular thickening of the pericardium and pleura bilateral (Figure 1a,b). The echocardiographic findings were consistent with constricting pericarditis. The inferior vena cava (IVC) was severely dilated with a central venous pressure (CVP) of 30 mm Hg. Intrahepatic venous dilation was also indicative of constrictive pericarditis (Figure 1c). There was marked septal shift with respiration and right ventricular compression (Figure 2a,b; see Additional file 1: Transthoracic echocardiogram showing marked interventricular movement.). Transthoracic echocardiogram (TTE) also showed restrictive movement of lateral ventricular walls with septal bounce (Figure 2c,d; see Additional file 2: Transthoracic echocardiogram showing restrictive movement of lateral ventricular walls with septal bounce). Mitral flow was decreased during inspiration, due to a reduced pressure gradient between the pulmonary vein and left atrium, and reduced left atrial filling (Figure 2E). As a result, the right atrium was significantly dilated.

Based on the CT findings, extensive malignancy or infection was suspected, as well as constrictive pericarditis. Examination of bronchial lavage and interventional biopsies failed to provide a definitive diagnosis. Therefore, pleural and pericardial biopsies were obtained by video-assisted thoracoscopic surgery. Dense adhesions and aged hematoma were found, and histopathological examination showed acute and chronic inflammation and fibrous thickening, with no evidence of malignancy. Results of serology testing for fungi, smears for acid-fast bacilli, culture for mycobacteria, and Gram staining were all negative, and white blood cells were rare in the biopsied tissues. These findings were consistent with pericarditis that was unlikely to be caused by microbial infection or immune disorder.

Selective cardiac catheterization, which revealed three-vessel coronary artery disease, was performed once extensive malignancy was excluded, and based on the results, we deemed a pericardiectomy was necessary. Our patient underwent emergency total pericardiectomy and triple coronary artery bypass. A standard median sternotomy was used for access and pericardiectomy performed off bypass. The pericardium was found to be grossly adherent, with thickening of up to 30 mm in some areas. Constricting layers of the epicardium were removed wherever possible. Evidence of an old hematoma was found throughout the diaphragmatic recess, and evidence of previous mediastinal haemorrhage was seen.

After the pericardiectomy, our patient's transesophageal echocardiographic findings showed an immediate response towards normalization, with resolution of tamponade. At the inferior cardiac-diaphragmatic sulcus, a large (60 mm), well-organized hematoma was entered and debrided. Cultures and gross specimens were sent for examination, and found to be negative for any infectious or oncologic source, consistent with the earlier findings. Heparinization and cardiopulmonary bypass was then initiated for saphenous vein grafting of the three coronary arteries found to have significant obstruction on catheterization. Our patient was weaned from bypass without complication on dobutamine 3 mg. He was extubated and stable within 12 hours of surgery. His post-operative recovery was unremarkable.

After recovery, our patient experienced improvement of all his previous symptoms. No further possible cause of his pericarditis was identified, except that on further conversation with our patient, he recalled falling and striking his anterior lower sternum and chest wall on the edge of a trailer hitch around 12-24 months previously. The accident had incapacitated him for several days, but he had not sought any medical therapy related to the trauma.

## Discussion

Diagnosis and treatment of constrictive pericarditis (CP) remains challenging. CP should be suspected in patients with clinical features of right-sides heart failure [4]. Other cardiac diseases must be excluded [2,3]. A previous history of pericarditis, open-heart surgery, tuberculosis, metastatic cancer and radiotherapy should be considered risk factors for developing CP. A significant percentage of patients diagnosed with CP do not have a known inciting cause. A previous history of chest trauma must be included in the differential diagnosis of CP.

The clinical diagnosis of CP relies primarily on appearance of edema and signs of cardiac insufficiency, such as dyspnea, upon physical examination. Non-invasive CT scan and echocardiography can greatly aid diagnosis. Pericarditis is associated with thickening of the pericardium, which may be visible on CT scans. CP is further associated with venous congestion, and dilation of the intrahepatic veins and inferior vena cava is readily seen by echocardiography. Reduced left atrial filling, which is the source of venous congestion, can be determined by echocardiography, and may be associated with inspiration [9]. Furthermore, abnormal septal movement is indicative of CP and can be seen by echocardiography. Accordingly, echocardiography should be performed at an early stage in patients presenting with symptoms associated with congestive heart failure, especially if CP is suspected.

Several cases involving the development of CP after chest trauma have been reported [5-8], but the exact pathogenesis of this specific initiation of CP is unknown. It has been suggested that development of CP after blunt trauma is dependent upon both damage to the mesothelium and the presence of blood in the pericardium [8]. The chronic presence of blood in the pericardium, caused by damage to blood vessels from blunt trauma is thought to gradually induce inflammation and tissue adhesions, resulting in cardiac tamponade.

Progression of CP after blunt trauma may occur at a relatively slow rate. It has been reported that the interval from the occurrence of blunt chest trauma to diagnosis of CP can range from three to 20 years [10]. This suggests that patients should be observed regularly after receiving blunt chest trauma to ensure early diagnosis of hemopericardium and resulting CP if either develop. In advanced cases, pericardiectomy is the definitive treatment for CP, and is recommended for most patients with a central venous pressure greater than 15 mm Hg [4]. The clinical course of constrictive pericarditis is usually progressive without surgical intervention.

## Conclusion

We report a case of trauma followed by progressive pericardial and pleural thickening. Subsequent development of chronic constrictive pericarditis occurred, requiring treatment by surgical pericardiectomy, as the clinical course of constrictive pericarditis is usually progressive without surgical intervention. Diagnosis of constrictive pericarditis remains challenging. Although uncommon, blunt trauma should be considered as a possible initiating cause for pericarditis. Delayed presentation of constrictive pericarditis should also be considered as a possible morbidity after blunt chest trauma. Our case also highlights the importance of performing echocardiography promptly in patients experiencing ongoing symptoms of congestive heart failure to allow earlier diagnosis of constrictive pericarditis or other cardiac disorder, and avoid unnecessary treatments.

## Patient's perspective

A little over a year before my heart surgery, I began having shortness of breath during daily exercise. During this time, my breathing problem became noticeably worse about six months before the operation, and my legs began to swell. One of my doctors gave me inhalers to help my breathing, but this didn't help. My shortness of breath got much worse about a couple of months before my operation. It became so bad that I would wake up in the middle of the night in a panic because I couldn't breathe, so I started sleeping in a chair. The swelling in my legs got so bad that my doctor thought I had an infection, but it was actually just fluid build-up in my feet.

After having seen my local physicians, I decided to visit the Mayo Clinic to see if they could help me. After talking with a few doctors, having a few tests done, and eventually having to go to the emergency room, I found out that the problem was with my heart. The doctors weren't sure exactly why, but the sac surrounding my heart had become hard and kept it from pumping correctly. Fortunately, my surgeons were able to remove the hardened tissue around my heart, and they also bypassed my coronary arteries before they became a problem. A few days after my operation, I returned home and started to feel better. I was on oxygen for a few weeks after my operation, but I quickly reached a point where I didn't need it any more. I am now able to live life with minimal restrictions on my physical activity. I am dancing and playing golf with my wife, and I am feeling very well. I am very grateful for the excellent care that I received at the Mayo Clinic and Hospital. I am certain that they saved my life.

## Competing interests

The authors declare that they have no competing interests.

## Consent

Written informed consent was obtained from our patient for publication of this case report and accompanying images. A copy of the written consent is available for review by the Editor-in-Chief of this journal.

## Authors' contributions

EMA reviewed clinical data, spoke with our patient, performed literature search, and wrote the final manuscript. DEJ and FAA interpreted clinical data, performed surgical intervention, guided manuscript development, and reviewed the manuscript. All authors read and approved the final manuscript.

## Supplementary Material

Additional file 1 **Transthoracic echocardiogram showing marked interventricular movement**. Click here for file

Additional file 2 **Transthoracic echocardiogram showing restrictive movement of lateral ventricular walls with septal bounce**. Click here for file
